# Supplementary material for: Multi‐dataset identification of innovative feature genes and molecular mechanisms in keratoconus
Source: J Cell Mol Med. 2024 Sep 19;28(18):e70079. doi: 10.1111/jcmm.70079 (PMC11412914; doi:10.1111/jcmm.70079)
Supplement: Supplementary file 2 — Material S2. [file JCMM-28-e70079-s002.zip › 7.GSEA analysis/5.RNF217-AS1/KEGG_NUCLEOTIDE_EXCISION_REPAIR.html]

Details for gene set KEGG\_NUCLEOTIDE\_EXCISION\_REPAIR[GSEA]

|  || Dataset | RNF217-AS1.group.cls#h\_versus\_l |
| Phenotype | group.cls#h\_versus\_l |
| Upregulated in class | l |
| GeneSet | KEGG\_NUCLEOTIDE\_EXCISION\_REPAIR |
| Enrichment Score (ES) | -0.33856633 |
| Normalized Enrichment Score (NES) | -1.4695624 |
| Nominal p-value | 0.01814516 |
| FDR q-value | 0.30249578 |
| FWER p-Value | 0.803 |
Table: GSEA Results Summary

  

Fig 1: Enrichment plot: KEGG\_NUCLEOTIDE\_EXCISION\_REPAIR      
 Profile of the Running ES Score & Positions of GeneSet Members on the Rank Ordered List

  

| SYMBOL | TITLE | RANK IN GENE LIST | RANK METRIC SCORE | RUNNING ES | CORE ENRICHMENT || 1 | RPA4 | na | 732 | 0.481 | 0.1227 | No |
| 2 | ERCC6 | na | 10198 | 0.083 | -0.1253 | No |
| 3 | POLE | na | 10398 | 0.080 | -0.1073 | No |
| 4 | ERCC5 | na | 11325 | 0.066 | -0.1144 | No |
| 5 | GTF2H1 | na | 12997 | 0.042 | -0.1500 | No |
| 6 | CDK7 | na | 13711 | 0.034 | -0.1605 | No |
| 7 | POLD4 | na | 14819 | 0.021 | -0.1861 | No |
| 8 | ERCC1 | na | 14933 | 0.020 | -0.1834 | No |
| 9 | RBX1 | na | 16317 | 0.006 | -0.2216 | No |
| 10 | POLD1 | na | 16368 | 0.005 | -0.2215 | No |
| 11 | XPA | na | 16706 | 0.002 | -0.2308 | No |
| 12 | CUL4B | na | 16841 | 0.000 | -0.2346 | No |
| 13 | RAD23A | na | 17441 | -0.006 | -0.2501 | No |
| 14 | RAD23B | na | 17977 | -0.011 | -0.2624 | No |
| 15 | POLE4 | na | 18097 | -0.012 | -0.2623 | No |
| 16 | GTF2H5 | na | 18316 | -0.014 | -0.2646 | No |
| 17 | CUL4A | na | 19597 | -0.026 | -0.2936 | No |
| 18 | CCNH | na | 19631 | -0.027 | -0.2867 | No |
| 19 | ERCC2 | na | 20642 | -0.036 | -0.3049 | No |
| 20 | POLD3 | na | 21754 | -0.048 | -0.3227 | Yes |
| 21 | MNAT1 | na | 22307 | -0.054 | -0.3225 | Yes |
| 22 | POLE3 | na | 22462 | -0.056 | -0.3103 | Yes |
| 23 | DDB2 | na | 22664 | -0.058 | -0.2988 | Yes |
| 24 | GTF2H3 | na | 22951 | -0.061 | -0.2887 | Yes |
| 25 | POLD2 | na | 23076 | -0.063 | -0.2735 | Yes |
| 26 | RPA1 | na | 23365 | -0.066 | -0.2620 | Yes |
| 27 | RFC5 | na | 23446 | -0.067 | -0.2442 | Yes |
| 28 | RFC1 | na | 23605 | -0.070 | -0.2279 | Yes |
| 29 | RPA2 | na | 23725 | -0.071 | -0.2100 | Yes |
| 30 | CETN2 | na | 23864 | -0.073 | -0.1921 | Yes |
| 31 | PCNA | na | 24885 | -0.089 | -0.1950 | Yes |
| 32 | RPA3 | na | 24916 | -0.089 | -0.1693 | Yes |
| 33 | LIG1 | na | 24995 | -0.090 | -0.1446 | Yes |
| 34 | ERCC8 | na | 25003 | -0.090 | -0.1178 | Yes |
| 35 | RFC2 | na | 25014 | -0.091 | -0.0911 | Yes |
| 36 | RFC4 | na | 25395 | -0.097 | -0.0731 | Yes |
| 37 | ERCC3 | na | 25722 | -0.102 | -0.0520 | Yes |
| 38 | ERCC4 | na | 26325 | -0.113 | -0.0356 | Yes |
| 39 | GTF2H4 | na | 27438 | -0.135 | -0.0273 | Yes |
| 40 | RFC3 | na | 27480 | -0.136 | 0.0122 | Yes |
| 41 | DDB1 | na | 27810 | -0.144 | 0.0457 | Yes |
| 42 | XPC | na | 28364 | -0.158 | 0.0768 | Yes |
| 43 | POLE2 | na | 32955 | -0.357 | 0.0511 | Yes |
Table: GSEA details [plain text format]

  

Fig 2: KEGG\_NUCLEOTIDE\_EXCISION\_REPAIR      
 Blue-Pink O' Gram in the Space of the Analyzed GeneSet

  

Fig 3: KEGG\_NUCLEOTIDE\_EXCISION\_REPAIR: Random ES distribution      
 Gene set null distribution of ES for **KEGG\_NUCLEOTIDE\_EXCISION\_REPAIR**
